# Supplementary material for: Unspecific post-mortem findings despite multiorgan viral spread in COVID-19 patients
Source: Crit Care. 2020 Aug 12;24:495. doi: 10.1186/s13054-020-03218-5 (PMC7422463; doi:10.1186/s13054-020-03218-5)
Supplement: Supplementary file 1 — Additional file 1. Critical care-autopsy-Covid. Additional material. Procedure to obtain brain samples. [file 13054_2020_3218_MOESM1_ESM.docx]

**Additional file 1: Additional Material**

Brain samples were obtained from 11 patients using a new procedure. A protection device was placed around the head of the patient (see figure below). Two openings were made in the device to allow the neurosurgical procedure. Right and left parieto-frontal drill holes were made using a hand-drill (to avoid use of an oscillating saw). The following samples were obtained from the right and left locations: frontal lobe en bloc resections and anterior and posterior stereotactic biopsies. Samples from the brainstem were obtained with a stereotactic needle. For each location, frozen and formalin-fixed paraffin-embedded (FFPE) samples were collected.

**Additional Figure S1:** Protection device placed around the head of the patient

**
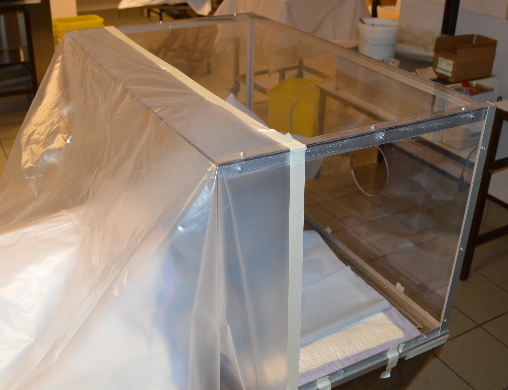
**
