# Supplementary material for: Unspecific post-mortem findings despite multiorgan viral spread in COVID-19 patients
Source: Crit Care. 2020 Aug 12;24:495. doi: 10.1186/s13054-020-03218-5 (PMC7422463; doi:10.1186/s13054-020-03218-5)
Supplement: Supplementary file 2 — Additional file 2: Critical care-autopsy-Covid. Additional Table S1. Laboratory findings on the day of admission. [file 13054_2020_3218_MOESM2_ESM.docx]

**Additional file 2: Table S1: Laboratory findings on the day of admission**

| ID | WBC  (*10^3^/mm³) | Lymphocytes  (*10^3^/mm³) | Hb  (g/dL) | Plt  (*10^3^/mm³) | CRP  (mg/L) | DD  (ng/mL) | LDH  (IU/L) | Troponin T hs  (ng/L) |
| --- | --- | --- | --- | --- | --- | --- | --- | --- |
| **1** | 8.58 | 0.43 | 12.3 | 174 | 10 | NA | 165 | 19 |
| **2** | 14.39 | 0.66 | 9.9 | 424 | 270 | NA | 1188 | 140 |
| **3** | 9.69 | 0.51 | 15.5 | 179 | 72 | NA | 840 | 32 |
| **4** | 7.93 | 1.20 | 11.7 | 465 | 86 | 1838 | 358 | 95 |
| **5** | 6.52 | 0.82 | 14.3 | 200 | 180 | NA | 393 | 11 |
| **6** | 6.94 | NA | 16.4 | 116 | 37 | 1066 | 521 | 39 |
| **7** | 9.65 | 2.11 | 11.8 | 194 | 270 | NA | 641 | 46 |
| **8** | 12.2 | 0.48 | 8.9 | 106 | 86 | NA | 259 | 235 |
| **9** | 8.69 | 0.70 | 11.4 | 200 | 170 | NA | 563 | 20 |
| **10** | 13.40 | 0.97 | 13.1 | 258 | 270 | 1789 | 802 | 26 |
| **11** | 5.28 | 0.59 | 11.4 | 131 | 150 | 1849 | 658 | 19 |
| **12** | 25.48 | 1.72 | 14.8 | 378 | 240 | NA | 1138 | 93 |
| **13** | 8.90 | 1.12 | 19.0 | 190 | 110 | 8531 | 640 | 46 |
| **14** | 4.67 | 0.62 | 13.5 | 142 | 27 | NA | 238 | 12 |
| **15** | 9.12 | 0.66 | 11.1 | 271 | 78 | 899 | 576 | 39 |
| **16** | 3.12 | 0.52 | 9.9 | 210 | 76 | 1319 | 319 | 24 |
| **17** | 7.02 | 0.83 | 9.1 | 220 | 130 | 2744 | 330 | 85 |

WBC: white blood cell count; Hb: hemoglobin concentration; Plt: Platelet count; CRP: C-reactive protein; DD: D-dimer; NA: not available; LDH: lactate dehydrogenase; hs: high sensitivity

The following pathogens were isolated during hospital/ICU stay: patient 5: *Proteus mirabilis*, *Enterobacter cloacae* (BAL); patient 6: *Klebsiella oxytoca* and *Aspergillus fumigatus* (BAL); patient 8: *Staphylococcus aureus* and *Bacteroides thetaiotaomicron* (blood culture); patient 9: *Enterobacter aerogenes* and *Staphylococcus aureus* (BAL); patient 12: *Enterobacter aerogenes* and *Escherichia coli* (BAL); patient 15: HSV1, candida, *Stenotrophomonas maltophilia*, *Staphylococcus aureus*, *Klebsiella oxytoca* and *Streptococcus pneumoniae* (BAL); patient 16: *Klebsiella pneumoniae*; patient 17: *Klebsiella pneumoniae* and *Streptococcus mitis* (BAL).
