# Supplementary material for: Unspecific post-mortem findings despite multiorgan viral spread in COVID-19 patients
Source: Crit Care. 2020 Aug 12;24:495. doi: 10.1186/s13054-020-03218-5 (PMC7422463; doi:10.1186/s13054-020-03218-5)
Supplement: Supplementary file 3 — Additional file 3: Critical care-autopsy-Covid. Additional Table S2. Detailed histological findings in all patients. [file 13054_2020_3218_MOESM3_ESM.docx]

**Additional file 3: Table S2: Detailed histological findings in all patients.**

| **ID** | **Lung** | **Other Organs** |
| --- | --- | --- |
| 1 | Bilateral lesions of E-DAD  Microthrombosis  Acute broncho-pneumonia (RIL, ML)  Acute pneumonia (LIL)  Atypical pneumocytes | **Cardiovascular system:**  Cardiomegaly  Ischemic cardiomyopathy  Fibrosis  Atheromatosis  **Gastro-intestinal system:**  Congestive hepatopathy  Esophagitis  Chronic gastritis  **Urinary system:**  Acute tubular injury (ATI) with severe autolysis  Hemosiderin granules in tubular epithelium  Pigmented casts  Arteriosclerosis  Discrete chronic cystitis  **CNS:**  Craniotomy for subdural hematoma drainage  Cerebral hemorrhage  Focal necrosis  Edema  Focal spongiosis  **Bone Marrow:**  Hyperplasia |
| 2 | Bilateral lesions of E-DAD  Bilateral lesions of L-DAD  Microthrombosis  Emphysema  Focal lympho-plasmocytic infiltrate  Atypical pneumocytes | **Cardiovascular system:**  Cardiomegaly  Ischemic cardiomyopathy  Fibrosis  Atheromatosis  **Gastrointestinal system:**  Congestive hepatopathy  Esophagitis  **Urinary system:**  Acute tubular injury (ATI) with severe autolysis  Pigmented casts  Severe arteriosclerosis  **Bone Marrow:**  Hyperplasia |
| 3 | E-DAD (L)  Microthrombosis  Emphysema  Left pneumonectomy | **Cardiovascular system:**  Cardiomegaly  Ischemic cardiomyopathy  Chronic pericarditis  Atheromatosis  **Gastrointestinal system:**  Congestive hepatopathy  Esophagitis  **Urinary system:**  Acute tubular injury (ATI) with severe autolysis  Hemosiderin granules in tubular epithelium  Pigmented casts  Severe arteriosclerosis  Chronic cystitis  **CNS:**  Vascular congestion  Extravasation of erythrocytes into the surrounding Virchow-Robin spaces  Hemosiderin-laden macrophages  Spongiosis (also observed in brainstem)  **Bone marrow:**  Hyperplasia with dysplastic megakaryocyte |
| 4 | Bilateral lesions of E-DAD  Microthrombosis  Emphysema  Focal lympho-plasmocytic infiltrate  Atypical pneumocytes | **Cardiovascular system:**  Ischemic cardiomyopathy  Pericardial effusion  Atheromatosis  **Gastrointestinal system:**  Ascites  Esophagitis  Peri-pancreatic steato-necrosis  Right adrenal carcinoma  **Urinary system:**  Acute tubular injury (ATI) with severe autolysis  Hemosiderin granules in tubular epithelium  Pigmented casts  Arteriosclerosis  **CNS:**  Brain cortical ischemic lesion with meningeal thickening associated to hemosiderin-laden macrophages  Cerebral hemorrhage  Spongiosis  **Bone marrow:**  Hyperplasia with dysplastic megakaryocyte |
| 5 | Bilateral lesions of E-DAD  L-DAD (RSL, LSL)  Microthrombosis  Acute broncho-pneumonia (RSL)  Lung infarct (RIL)  Right bilobectomy (RIL-ML) | **Cardiovascular system:**  Cardiomegaly  Ischemic cardiomyopathy  Pericardial effusion  Atheromatosis  **Gastrointestinal system:**  Hepatomegaly  Hepatic steatosis (40%)  Cholestasis  Macroscopic and microscopic ischemic enteritis associated with microthrombi  **Urinary system:**  ATI not assessable due to severe autolysis  Hemorrhagic cystitis  **CNS:**  Edema  Hemorrhagic suffusion  Spongiosis (also located to brainstem and centrum ovale)  **Bone marrow:**  Hyperplasia with dysplastic megakaryocyte |
| 6 | Bilateral lesions of E-DAD  Bilateral lesions of L-DAD  Microthrombosis  Invasive aspergillosis (lung and trachea)  Acute broncho-pneumonia (LIL)  Acute pneumonia (LIL, ML, RSL, RIL)  Atypical pneumocytes | **Cardiovascular system:**  Recent coronary stent  Cardiomegaly  Ischemic cardiomyopathy  Fibrosis  Severe atheromatosis  Abdominal aortic aneurysm  **Gastrointestinal system:**  Hepatomegaly  Hepatic steatosis (<5%)  Esophagitis with few multinucleated giant cells  Gastric congestion  **Urinary system:**  ATI not assessable due to severe autolysis  Hemosiderin granules in tubular epithelium  Pigmented casts  Moderate arteriosclerosis  **Bone marrow:**  Hyperplasia |
| 7 | Bilateral lesions of E-DAD  L-DAD (LSL)  Bilateral invasive aspergillosis  Acute broncho-pneumonia (RIL)  Acute pneumonia (RIL,ML)  Inhalation pneumonia  Lung infarct (LIL) | **Cardiovascular system:**  Ischemic cardiomyopathy  **Gastrointestinal system:**  Hepatic steatosis (50%)  Esophagitis  Gastric congestion  Splenic white pulp hypoplasia  **Urinary system:**  ATI not assessable due to severe autolysis  Hemosiderin granules in tubular epithelium  Pigmented casts  Discrete arteriosclerosis |
| 8 | Bilateral lesions of E-DAD  Acute pneumonia (RSL)  Advanced interstitial fibrosis  Pulmonary hypertension pneumopathy  Emphysema | **Cardiovascular system:**  Recent coronary stent  Ischemic cardiomyopathy  Severe atheromatosis  **Gastrointestinal system:**  Congestive hepatopathy  Esophagitis  Gastric congestion  Colic sevelamer crystals  Chronic pancreatitis  **Urinary system:**  ATI not assessable due to severe autolysis  Thrombus in an interlobar artery  Hemosiderin granules in tubular epithelium  Pigmented casts  Diabetic nephropathy  Severe chronic pyelonephritis  Calcium oxalate crystal deposits  Severe arteriosclerosis  Chronic cystitis  **CNS:**  Edema  Focal necrosis  Spongiosis  **Bone marrow:**  Hyperplasia with dysplastic megakaryocytes |
| 9 | Bilateral lesions of E-DAD  L-DAD (ML)  Microthrombosis  Lung infarct  Inhalation pneumonia | **Cardiovascular system:**  Cardiomegaly  Ischemic cardiomyopathy  Fibrosis  Discrete atheromatosis  **Gastrointestinal system:**  Hepatic steatosis (<5%)  Kayexalate crystals in the esophagus, stomach and colon  Adrenal cortical adenoma  **Urinary system:**  ATI not assessable due to severe autolysis  Hemosiderin granules in tubular epithelium  Pigmented casts  Chronic interstitial inflammation  Severe arteriosclerosis  Hemorrhagic cystitis  **CNS:**  Edema  Focal necrosis  Hemorrhagic suffusion  Spongiosis (located to brain and brainstem)  **Bone marrow:**  Hyperplasia with dysplastic megakaryocyte |
| 10 | Bilateral lesions of E-DAD  L-DAD (L)  Microthrombosis  Hyperplasia of pneumocytes type-II  Syncytial multinucleated giant cells | **Cardiovascular system:**  Cardiomegaly  Ischemic cardiomyopathy  **Gastrointestinal system:**  Hepatomegaly, congestive hepatopathy  Hepatic steatosis <5%  Chronic hepatitis  Kayexalate crystals in the stomach and colon  **Urinary system:**  ATI not assessable due to severe autolysis  Calcium deposits in the medulla  Severe arteriosclerosis  **CNS:**  Hemorrhagic suffusion  Spongiosis |
| 11 | Bilateral lesions of E-DAD  Hyperplasia of pneumocytes type-II  Syncytial multinucleated giant cells | **Cardiovascular system:**  Cardiomegaly  Ischemic cardiomyopathy  **Gastrointestinal system:**  Liver cirrhosis  Hepatic steatosis <5%  Microsphere bead for hepatocarcinoma  Esophagitis  **Urinary system:**  ATI not assessable due to severe autolysis  Diabetic nephropathy  Discrete glomerulosclerosis  Chronic pyelonephritis  Pigmented casts  Calcium deposits in medulla  Moderate arteriosclerosis  Prostatic adenocarcinoma  **Bone Marrow:**  Hyperplasia |
| 12 | Bilateral lesions of E-DAD  L-DAD (L)  Microthrombosis  Pulmonary embolism (RIL)  Bronchitis (LIL, ML)  Acute pneumonia (LSL, RIL)  Acute broncho-pneumonia (ML)  Syncytial multinucleated giant cells | **Cardiovascular system:**  Cardiomegaly  Ischemic cardiomyopathy  **Gastrointestinal system:**  Hepatomegaly  Hepatic steatosis (5%)  Esophagitis  Kayexalate crystals in the esophagus, stomach and colon  **Urinary system:**  ATI not assessable due to severe autolysis  Hemosiderin granules in tubular epithelium  Pigmented casts  Discrete glomerulosclerosis  Chronic pyelonephritis  Moderate arteriosclerosis  Subacute cystitis.  **CNS:**  Hemorrhagic suffusion  Spongiosis  Extravasation of erythrocytes into the surrounding Virchow-Robin spaces  Hemosiderin-laden macrophages  **Bone Marrow:**  Hyperplasia |
| 13 | Bilateral lesions of E-DAD  Perivascular chronic inflammatory infiltrate  Acute broncho-pneumonia (LIL) | **Cardiovascular system:**  Cardiomegaly  Ischemic cardiomyopathy  Fibrosis  **Gastrointestinal system:**  Liver cirrhosis  Chronic hepatitis  Hepatic steatosis (5%)  Barrett’s esophagus  Discrete gastritis  **Urinary system:**  ATI not assessable due to severe autolysis  Moderate glomerulosclerosis  Chronic pyelonephritis  Discrete arteriosclerosis.  Follicular cystitis  **Bone Marrow:**  Hyperplasia (predominantly myeloid) |
| 14 | Bilateral alveolar edema  Bilateral aspiration pneumonia  Perivascular chronic inflammatory infiltrate | **Cardiovascular system:**  Cardiomegaly  Ischemic cardiomyopathy  **Gastrointestinal system:**  Congestive hepatopathy  Esophagitis  **Urinary system:**  ATI not assessable due to autolysis  Hyaline arteriosclerosis  Angiomyolipoma (2mm)  **Bone Marrow:**  Hyperplasia |
| 15 | Bilateral E-DAD  L-DAD (L)  Microthrombosis  Lung infarct (RIL, LIL ,RSL,ML)  Pulmonary embolism (L) | **Cardiovascular system:**  Cardiomegaly  Recent myocardial infarction  **Gastrointestinal system:**  Hepatomegaly  Hepatic steatosis (10%)  Esophagitis  Gastritis  **Urinary system:**  ATI not assessable due to severe autolysis  Pigmented casts  Discrete arteriosclerosis.  Acute mycotic cystitis  Vascular thrombi into the bladder  **CNS:**  Hemorrhagic suffusion  Hemosiderin-laden macrophages  Extravasation of erythrocytes into the surrounding Virchow-Robin spaces |
| 16 | Bilateral E-DAD  L-DAD (L)  Microthrombosis  Hyperplasia of pneumocytes type-II | **Cardiovascular system:**  Cardiomegaly  Recent myocardial infarction  **Gastrointestinal system:**  Hepatic steatosis (5%)  Centro-lobular hepatic necrosis  Esophagitis  Gastritis  **Kidneys and urinary system:**  ATI not assessable due to severe autolysis  Hemosiderin granules in tubular epithelium  Pigmented casts  Moderate glomerulosclerosis  Moderate arteriosclerosis  Subacute cystitis  **CNS:**  Spongiosis  Extravasation of erythrocytes into the surrounding Virchow-Robin spaces  Hemosiderin-laden macrophages  Edema  **Bone Marrow:**  Hyperplasia  **Others:**  Thyroid follicular carcinoma |
| 17 | Bilateral hemorrhage (post-thoracotomy) Embolization right bronchial artery  L-DAD  Acute broncho-pneumonia (LSL; LIL) | **Cardiovascular system:**  Cardiomegaly  Ischemic cardiomyopathy  **Gastrointestinal system, liver:**  Congestive hepatopathy  Chronic hepatitis (C-virus)  Esophagitis  Gastritis  Acute pancreatitis  **Urinary system:**  Acquired cystic kidney disease (ACKD)  Multiple tubular adenomas in native kidneys  ATI not assessable due to severe autolysis in kidney transplant  **CNS:**  Spongiosis  Hemorrhagic suffusion  Hemosiderin-laden macrophages  **Bone Marrow:**  Hyperplasia |

E-DAD: Diffuse alveolar damage early phase, L-DAD: Diffuse alveolar damage late phase, R: right lung, L: left lung, RSL: right superior lobe, ML: right middle lobe, RIL: right superior lobe, LSL: left superior lobe, LIL: left inferior lobe, ATI: acute tubular injury; CNS: central nervous system
